# Bis(μ-cobaltoceniumseleno­late-1:2κ^2^ *Se*:*Se*)bis[bis­(cobaltoceniumseleno­late-κ*Se*)mercury(II)] tetra­kis­(hexa­fluorido­phosphate) aceto­nitrile disolvate

**DOI:** 10.1107/S241431462101083X

**Published:** 2021-10-21

**Authors:** Daniel Menia, Klaus Wurst, Benno Bildstein

**Affiliations:** a University of Innsbruck, Faculty of Chemistry and Pharmacy, Innrain 80-82, 6020 Innsbruck, Austria; Vienna University of Technology, Austria

**Keywords:** cobalt, selenium, mercury, sandwich complexes, crystal structure

## Abstract

The title compound, [Co_6_Hg_2_(C_5_H_5_)_6_(C_5_H_4_Se)_6_](PF_6_)_4_·2CH_3_CN or [Hg_2_(CcSe)_6_][PF_6_]_4_·2CH_3_CN (Cc = C_10_H_9_Co), was obtained as bright-orange needle-shaped crystals. It is a salt containing a tetra­cationic dimercury species with six cobaltoceniumseleno­late ligands, four hexa­fluorido­phosphate counter-ions and two aceto­nitrile solvent mol­ecules. The cation (point group  ) has a bi­tetra­hedral {Hg_2_Se_6_} core with two bridging Se atoms and four terminal Se atoms.

## Abstract 2

The cation in the title salt has a bi­tetra­hedral {Hg_2_Se_6_} core.

## Graphical abstract

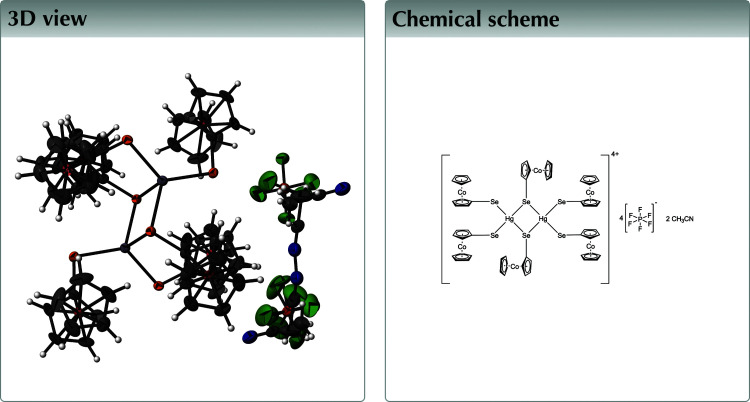

## Structure description

Zwitterionic cobaltoceniumseleno­late is a versatile ligand for forming organometallic coordination compounds (Vanicek *et al.*, 2018 ▸). The title salt, [Hg_2_(CcSe)_6_][PF_6_]_4_·2CH_3_CN (Cc = C_10_H_9_Co), was synthesized starting from the recently reported cobaltocenium seleno­late gold(I) tri­phenyl­phosphine hexa­fluorido­phosphate (Menia *et al.*, 2021 ▸) using elemental mercury in dry *ortho*-dichlorbenzene. It was crystallized as an aceto­nitrile solvate showing positional disorder of the solvent mol­ecule and of one of the PF_6_^−^ anions.

**Figure 1:**
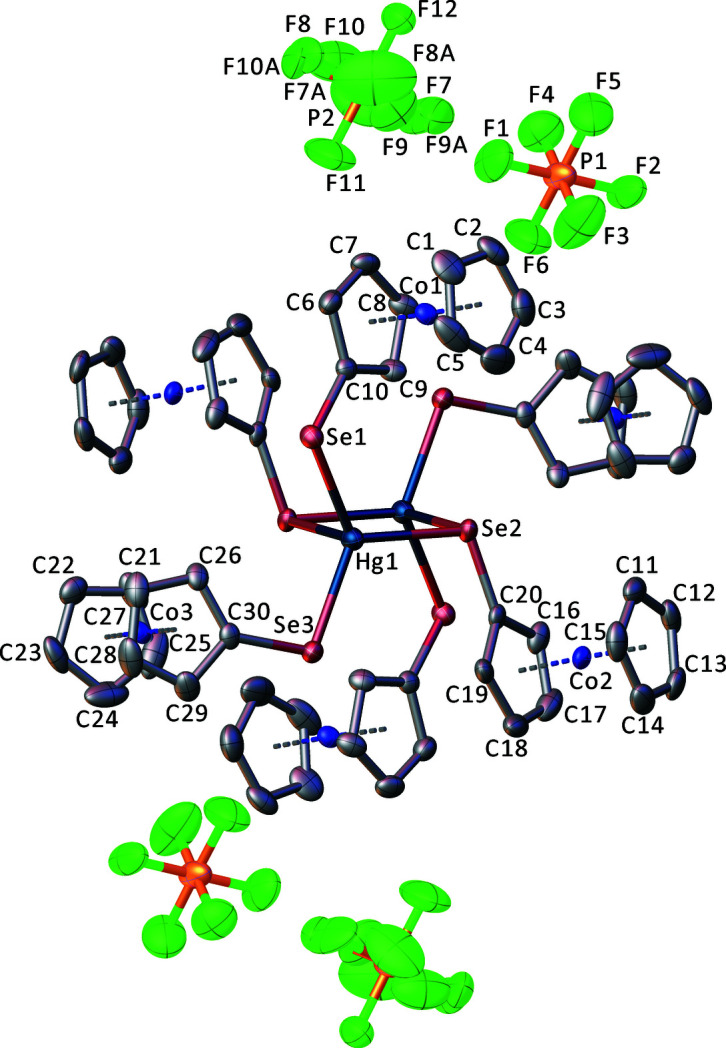
The mol­ecular entities of the title salt, with displacement ellipsoids drawn at the 50% probability level. Hydrogen atoms and solvent mol­ecules were omitted for clarity. Non-labeled atoms are generated by symmetry operation −*x* + 1, −*y* + 1, −*z* + 1.

The cation lies about a crystallographic inversion center and has a bi­tetra­hedral {Hg_2_Se_6_} core formed by edge-sharing of two HgSe_4_ tetra­hedra and has two bridging Se2 atoms and four terminal Se1 and Se3 atoms (Fig. 1 ▸). The Se2—Hg1—Se2^i^ angle between the bridging Se atoms is 91.509 (10)°, resulting in an Hg1—Se1—Hg1^i^ angle of 88.491 (10)° [symmetry code: (i) –*x* + 1, –*y* + 1, –*z* + 1]. The four C_*ipso*_—Se—Hg1 angles are slightly compressed, ranging from 98.79 (7) to 106.04 (7)°, as was also observed for cobaltocenium seleno­late gold complexes (Menia *et al.*, 2021 ▸). The terminal Se—Hg1 bond lengths are 2.5476 (3) (Se1—Hg1) and 2.5451 (3) Å (Se3—Hg1), whereas the bridging Se2—Hg1 bond lengths differ considerably with 2.6254 (3) Å for Se2—Hg1 and 3.1537 (4) for Se2—Hg1^i^. With an average Se—C distance of 1.89 Å between the seleno­late and the cobaltocenium residues, these bond lengths are comparable with other recently reported cobaltocenium seleno­lates (Menia *et al.*, 2021 ▸).

The sole comparable compound found in the literature is [Yb(C_4_H_10_O_2_)_4_][Hg_2_(C_6_H_5_Se)_6_] (Romanelli *et al.*, 2008 ▸). Here, instead of the cationic cobaltocenium species the selenium atoms are bonded to phenyl residues, which makes the Hg species a dianion. With an average Se—Hg bond length of 2.81 Å in the title compound, bonds are elongated in comparison with the dianion of Romanelli *et al.* (2.68 Å).

**Figure 2:**
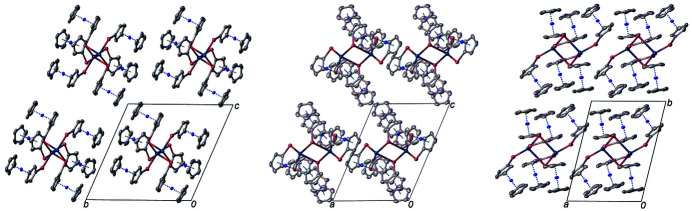
Mol­ecular packing in the title compound with displacement ellipsoids drawn at the 50% probability level in views along the *a* (left)*, b* (center) and *c* (right) axes. Hydrogen atoms, anions and solvent mol­ecules are omitted for clarity.

Since the packing of the mol­ecules (Fig. 2 ▸) shows no remarkable hydrogen bonding or *π*-stacking inter­actions, the cohesion within the crystal structure is dominated by van der Waals forces.

## Synthesis and crystallization

In a 50 ml Schlenk flask, 11.1 mg of [(CcSe)(PPh_3_)Au]PF_6_ (1 eq., 0.013 mmol) were suspended in 5 ml of dry *ortho*-di­chloro­benzene. Approximately 0.1 ml of liquid mercury was added and the mixture stirred for 48 h. This reaction was originally carried out with the aim of removing selenium from the desired compound. The bright-orange precipitate was filtered off, washed with two portions of 10 ml of diethyl ether and dissolved in 5 ml of aceto­nitrile. This orange solution was concentrated to about 1 ml. Bright-orange needle-shaped crystals were obtained by diffusion-crystallization with diethyl ether at 253 K. ^1^H NMR (300 MHz, CD_3_CN): δ 5.70 (*t*, *J* = 2.0 Hz, 2H), 5.51 (*t*, *J* = 2.0 Hz, 2H), 5.46 (*s*, 5H).

## Refinement

Crystal data, data collection and structure refinement details are summarized in Table 1 ▸. One of the two PF_6_ anions (P2) shows disorder of four fluorine atoms over two sets of sites in a 2:1 ratio for F7, F8, F9, F10 and F7*A*, F8*A*, F9*A*, F10*A*. Another positional disorder occurs for the complete aceto­nitrile solvent mol­ecule in a 1:1 ratio. All disordered atoms were refined with anisotropic displacement parameters without further restraints, but with fixed occupation factors.

## Supplementary Material

Crystal structure: contains datablock(s) global, I. DOI: 10.1107/S241431462101083X/wm4154sup1.cif

Structure factors: contains datablock(s) I. DOI: 10.1107/S241431462101083X/wm4154Isup2.hkl

CCDC reference: 2116450

Additional supporting information:  crystallographic information; 3D view; checkCIF report

## Figures and Tables

**Table 1:** Experimental details

Crystal data	
Chemical formula	[Co_6_Hg_2_(C_5_H_5_)_6_(C_5_H_4_Se)_6_](PF_6_)_4_·2C_2_H_3_N
*M*_r_	2665.54
Crystal system, space group	Triclinic, *P* 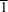
Temperature (K)	183
*a*, *b*, *c* (Å)	10.2933 (8), 14.3271 (12), 15.1087 (12)
α, β, γ (°)	109.744 (3), 109.764 (2), 95.306 (3)
*V* (Å^3^)	1919.1 (3)
*Z*	1
Radiation type	Mo *K*α
μ (mm^−1^)	8.28
Crystal size (mm)	0.18 × 0.09 × 0.04

Data collection	
Diffractometer	Bruker D8 QUEST PHOTON 100
Absorption correction	Multi-scan (*SADABS*; Krause *et al.*, 2015 ▸)
*T*_min_, *T*_max_	0.574, 0.837
No. of measured, independent and observed [*I* > 2σ(*I*)] reflections	69321, 7546, 7005
*R*_int_	0.038
(sin θ/λ)_max_ (Å^−1^)	0.617

Refinement	
*R*[*F* ^2^ > 2σ(*F* ^2^)], *wR*(*F* ^2^), *S*	0.017, 0.042, 1.04
No. of reflections	7546
No. of parameters	553
H-atom treatment	H-atom parameters constrained
Δρ_max_, Δρ_min_ (e Å^−3^)	0.77, −0.80

Computer programs: *APEX2* and *SAINT* (Bruker, 2014 ▸), *SHELXT* (Sheldrick, 2015*a* ▸), *SHELXL* (Sheldrick, 2015*b* ▸), *OLEX2* (Dolomanov *et al.*, 2009 ▸) and *publCIF* (Westrip, 2010 ▸).

## full crystallographic data

### Crystal data

**Table:**

[Co_6_Hg_2_(C_5_H_5_)_6_(C_5_H_4_Se)_6_](PF_6_)_4_·2C_2_H_3_N	*Z* = 1
*M**_r_* = 2665.54	*F*(000) = 1260
Triclinic, *P*1	*D*_x_ = 2.306 Mg m^−^^3^
*a* = 10.2933 (8) Å	Mo *K*α radiation, λ = 0.71073 Å
*b* = 14.3271 (12) Å	Cell parameters from 9672 reflections
*c* = 15.1087 (12) Å	θ = 2.3–26.4°
α = 109.744 (3)°	µ = 8.28 mm^−^^1^
β = 109.764 (2)°	*T* = 183 K
γ = 95.306 (3)°	Prism, orange
*V* = 1919.1 (3) Å^3^	0.18 × 0.09 × 0.04 mm

### Data collection

**Table:**

Bruker D8 QUEST PHOTON 100 diffractometer	7546 independent reflections
Radiation source: Incoatec Microfocus	7005 reflections with *I* > 2σ(*I*)
Multi layered optics monochromator	*R*_int_ = 0.038
Detector resolution: 10.4 pixels mm^-1^	θ_max_ = 26.0°, θ_min_ = 2.2°
φ and ω scans	*h* = −12→12
Absorption correction: multi-scan (SADABS; Krause *et al.*, 2015)	*k* = −17→17
*T*_min_ = 0.574, *T*_max_ = 0.837	*l* = −18→18
69321 measured reflections	

### Refinement

**Table:**

Refinement on *F*^2^	Hydrogen site location: inferred from neighbouring sites
Least-squares matrix: full	H-atom parameters constrained
*R*[*F*^2^ > 2σ(*F*^2^)] = 0.017	*w* = 1/[σ^2^(*F*_o_^2^) + (0.0197*P*)^2^ + 1.8079*P*] where *P* = (*F*_o_^2^ + 2*F*_c_^2^)/3
*wR*(*F*^2^) = 0.042	(Δ/σ)_max_ = 0.003
*S* = 1.04	Δρ_max_ = 0.77 e Å^−^^3^
7546 reflections	Δρ_min_ = −0.80 e Å^−^^3^
553 parameters	Extinction correction: SHELXL2014/7 (Sheldrick, 2015b), Fc^*^=kFc[1+0.001xFc^2^λ^3^/sin(2θ)]^-1/4^
0 restraints	Extinction coefficient: 0.00230 (10)
Primary atom site location: structure-invariant direct methods	

### Special details

**Table:**

Geometry. All esds (except the esd in the dihedral angle between two l.s. planes) are estimated using the full covariance matrix. The cell esds are taken into account individually in the estimation of esds in distances, angles and torsion angles; correlations between esds in cell parameters are only used when they are defined by crystal symmetry. An approximate (isotropic) treatment of cell esds is used for estimating esds involving l.s. planes.
Refinement. C-bound hydrogen atoms were placed in calculated positions and constrained to ride on their parent atoms with *U*_iso_(H) = 1.2*U*_eq_(C) and a C—H distance of 0.95 Å for aromatic H atoms. Positinal disorder of same flourine atoms at P2 in ratio 2:1 for F7-F10 : F7a-F10a and for solvent acetonitrile C32-C31-N1 : C32a-C31a-N1a in ratio 1:1, respectively.

### Fractional atomic coordinates and isotropic or equivalent isotropic displacement parameters (Å^2^)

**Table:**

	*x*	*y*	*z*	*U*_iso_*/*U*_eq_	Occ. (<1)
Hg1	0.31028 (2)	0.47867 (2)	0.51180 (2)	0.02781 (4)	
Se1	0.32480 (3)	0.45328 (2)	0.67355 (2)	0.02603 (6)	
Se2	0.40933 (3)	0.35008 (2)	0.39551 (2)	0.02572 (6)	
Se3	0.13272 (3)	0.56435 (2)	0.42414 (2)	0.02556 (6)	
Co1	0.51168 (3)	0.26149 (2)	0.69145 (3)	0.02302 (8)	
Co2	0.21259 (4)	0.23319 (2)	0.12527 (3)	0.02438 (8)	
Co3	0.18455 (3)	0.79863 (2)	0.63293 (3)	0.02143 (7)	
C1	0.4091 (3)	0.1559 (2)	0.7209 (3)	0.0462 (8)	
H1	0.4018	0.1660	0.7843	0.055*	
C2	0.5175 (3)	0.1204 (2)	0.6918 (3)	0.0416 (7)	
H2	0.5954	0.1018	0.7320	0.050*	
C3	0.4906 (4)	0.1174 (2)	0.5936 (3)	0.0482 (8)	
H3	0.5471	0.0967	0.5554	0.058*	
C4	0.3634 (4)	0.1508 (2)	0.5614 (3)	0.0580 (11)	
H4	0.3197	0.1566	0.4977	0.070*	
C5	0.3142 (3)	0.1739 (2)	0.6405 (3)	0.0519 (10)	
H5	0.2308	0.1976	0.6395	0.062*	
C6	0.5531 (3)	0.3958 (2)	0.8092 (2)	0.0312 (6)	
H6	0.5136	0.4081	0.8594	0.037*	
C7	0.6793 (3)	0.3613 (2)	0.8153 (2)	0.0337 (6)	
H7	0.7394	0.3473	0.8703	0.040*	
C8	0.6998 (3)	0.3516 (2)	0.7255 (2)	0.0312 (6)	
H8	0.7763	0.3297	0.7093	0.037*	
C9	0.5863 (3)	0.38023 (19)	0.6631 (2)	0.0267 (5)	
H9	0.5737	0.3801	0.5978	0.032*	
C10	0.4948 (3)	0.40899 (17)	0.71494 (19)	0.0233 (5)	
C11	0.1956 (3)	0.0996 (2)	0.1453 (2)	0.0393 (7)	
H11	0.2363	0.0915	0.2080	0.047*	
C12	0.2595 (3)	0.0951 (2)	0.0760 (2)	0.0402 (7)	
H12	0.3511	0.0831	0.0835	0.048*	
C13	0.1648 (4)	0.1113 (2)	−0.0067 (2)	0.0411 (7)	
H13	0.1816	0.1126	−0.0644	0.049*	
C14	0.0411 (3)	0.1252 (2)	0.0110 (2)	0.0438 (8)	
H14	−0.0407	0.1371	−0.0328	0.053*	
C15	0.0598 (3)	0.1183 (2)	0.1054 (3)	0.0437 (8)	
H15	−0.0069	0.1250	0.1365	0.052*	
C16	0.3985 (3)	0.34072 (19)	0.1954 (2)	0.0338 (6)	
H16	0.4904	0.3277	0.2072	0.041*	
C17	0.3071 (4)	0.3502 (2)	0.1062 (2)	0.0482 (9)	
H17	0.3276	0.3454	0.0483	0.058*	
C18	0.1804 (4)	0.3679 (2)	0.1184 (2)	0.0474 (9)	
H18	0.1010	0.3775	0.0702	0.057*	
C19	0.1920 (3)	0.36898 (19)	0.2153 (2)	0.0323 (6)	
H19	0.1211	0.3780	0.2425	0.039*	
C20	0.3288 (3)	0.35406 (17)	0.26440 (19)	0.0241 (5)	
C21	0.3979 (3)	0.8491 (2)	0.6884 (3)	0.0446 (8)	
H21	0.4663	0.8112	0.7061	0.053*	
C22	0.3452 (3)	0.9115 (2)	0.7541 (2)	0.0368 (7)	
H22	0.3713	0.9237	0.8243	0.044*	
C23	0.2474 (3)	0.9533 (2)	0.6989 (3)	0.0456 (8)	
H23	0.1953	0.9988	0.7249	0.055*	
C24	0.2399 (4)	0.9162 (3)	0.5986 (3)	0.0642 (12)	
H24	0.1818	0.9318	0.5442	0.077*	
C25	0.3341 (4)	0.8515 (3)	0.5931 (3)	0.0603 (12)	
H25	0.3509	0.8156	0.5339	0.072*	
C26	0.1367 (3)	0.65878 (18)	0.6361 (2)	0.0279 (6)	
H26	0.2027	0.6255	0.6669	0.033*	
C27	0.0690 (3)	0.7278 (2)	0.6860 (2)	0.0373 (7)	
H27	0.0805	0.7476	0.7554	0.045*	
C28	−0.0186 (3)	0.7617 (2)	0.6146 (3)	0.0439 (8)	
H28	−0.0767	0.8081	0.6275	0.053*	
C29	−0.0046 (3)	0.7144 (2)	0.5202 (2)	0.0350 (6)	
H29	−0.0500	0.7252	0.4594	0.042*	
C30	0.0896 (3)	0.64768 (18)	0.5321 (2)	0.0242 (5)	
P1	0.94494 (10)	0.11818 (8)	0.70615 (7)	0.0506 (2)	
F1	0.9016 (3)	0.1795 (2)	0.79546 (19)	0.0935 (9)	
F2	0.9873 (3)	0.0561 (2)	0.61544 (18)	0.0837 (8)	
F3	0.7858 (3)	0.0741 (3)	0.6318 (2)	0.1244 (14)	
F4	1.1077 (3)	0.1638 (2)	0.7786 (2)	0.0832 (7)	
F5	0.9538 (3)	0.0274 (2)	0.7437 (2)	0.0931 (9)	
F6	0.9436 (4)	0.2112 (3)	0.6708 (3)	0.1183 (12)	
P2	0.65318 (11)	0.20499 (8)	1.02043 (7)	0.0507 (2)	
F11	0.5820 (5)	0.2828 (3)	0.9804 (3)	0.1310 (14)	
F12	0.7338 (3)	0.13036 (18)	1.06292 (17)	0.0688 (6)	
N1	0.6305 (9)	0.5411 (7)	1.0496 (6)	0.077 (2)	0.5
C31	0.7429 (13)	0.5419 (11)	1.0966 (8)	0.058 (3)	0.5
C32	0.8832 (15)	0.5370 (17)	1.1525 (14)	0.077 (4)	0.5
H32A	0.8795	0.4739	1.1644	0.115*	0.5
H32B	0.9248	0.5957	1.2183	0.115*	0.5
H32C	0.9415	0.5381	1.1131	0.115*	0.5
N1A	0.9757 (6)	0.5167 (5)	1.0373 (5)	0.0523 (14)	0.5
C31A	0.9058 (7)	0.5274 (6)	1.0817 (5)	0.0459 (15)	0.5
C32A	0.8167 (17)	0.5410 (13)	1.1386 (14)	0.087 (6)	0.5
H32D	0.7207	0.4990	1.0938	0.130*	0.5
H32E	0.8132	0.6129	1.1648	0.130*	0.5
H32F	0.8556	0.5204	1.1961	0.130*	0.5
F7	0.6905 (9)	0.1622 (8)	0.9240 (5)	0.118 (3)	0.67
F8	0.6295 (11)	0.2495 (9)	1.1213 (8)	0.082 (3)	0.67
F9	0.5086 (7)	0.1332 (5)	0.9654 (5)	0.0956 (18)	0.67
F10	0.8129 (6)	0.2804 (5)	1.0832 (6)	0.105 (2)	0.67
F7A	0.7705 (17)	0.2628 (12)	1.0115 (16)	0.121 (5)	0.33
F8A	0.5165 (16)	0.1357 (19)	1.0199 (17)	0.168 (9)	0.33
F9A	0.609 (2)	0.1161 (9)	0.9079 (10)	0.121 (7)	0.33
F10A	0.668 (3)	0.281 (2)	1.1255 (19)	0.154 (13)	0.33

### Atomic displacement parameters (Å^2^)

**Table:**

	*U*^11^	*U*^22^	*U*^33^	*U*^12^	*U*^13^	*U*^23^
Hg1	0.03160 (6)	0.02915 (6)	0.02704 (6)	0.01267 (4)	0.01398 (4)	0.01231 (4)
Se1	0.02882 (13)	0.02585 (13)	0.02935 (14)	0.00876 (10)	0.01531 (11)	0.01336 (11)
Se2	0.02821 (13)	0.02356 (13)	0.02013 (13)	0.00917 (10)	0.00767 (10)	0.00348 (10)
Se3	0.02908 (13)	0.02367 (12)	0.02107 (13)	0.00690 (10)	0.00785 (10)	0.00730 (10)
Co1	0.02233 (16)	0.01826 (16)	0.02694 (18)	0.00221 (12)	0.00697 (14)	0.01050 (14)
Co2	0.02932 (18)	0.01654 (16)	0.01857 (17)	0.00271 (13)	0.00503 (14)	0.00182 (13)
Co3	0.02007 (16)	0.01581 (15)	0.02571 (18)	0.00371 (12)	0.00727 (14)	0.00689 (13)
C1	0.0466 (18)	0.0297 (15)	0.071 (2)	−0.0005 (13)	0.0282 (17)	0.0274 (16)
C2	0.0366 (16)	0.0237 (14)	0.064 (2)	0.0061 (12)	0.0136 (15)	0.0233 (14)
C3	0.057 (2)	0.0213 (14)	0.054 (2)	0.0030 (13)	0.0219 (17)	0.0022 (14)
C4	0.059 (2)	0.0277 (16)	0.047 (2)	−0.0104 (15)	−0.0151 (18)	0.0080 (15)
C5	0.0243 (15)	0.0290 (16)	0.090 (3)	−0.0009 (12)	0.0104 (17)	0.0234 (17)
C6	0.0413 (15)	0.0252 (13)	0.0229 (13)	0.0037 (11)	0.0104 (12)	0.0079 (11)
C7	0.0294 (14)	0.0290 (14)	0.0305 (15)	−0.0014 (11)	−0.0020 (12)	0.0132 (12)
C8	0.0223 (13)	0.0301 (14)	0.0419 (16)	0.0020 (10)	0.0085 (12)	0.0202 (13)
C9	0.0259 (13)	0.0285 (13)	0.0318 (14)	0.0039 (10)	0.0129 (11)	0.0180 (11)
C10	0.0254 (12)	0.0158 (11)	0.0250 (13)	0.0003 (9)	0.0073 (10)	0.0072 (10)
C11	0.0577 (19)	0.0170 (13)	0.0280 (15)	−0.0002 (12)	0.0064 (14)	0.0039 (11)
C12	0.0337 (15)	0.0198 (13)	0.0475 (19)	0.0063 (11)	0.0087 (14)	−0.0024 (12)
C13	0.0564 (19)	0.0258 (14)	0.0254 (15)	0.0006 (13)	0.0142 (14)	−0.0041 (12)
C14	0.0368 (16)	0.0258 (14)	0.0384 (18)	0.0021 (12)	−0.0049 (14)	−0.0017 (13)
C15	0.0432 (17)	0.0223 (14)	0.052 (2)	−0.0044 (12)	0.0247 (15)	−0.0040 (13)
C16	0.0404 (16)	0.0215 (13)	0.0314 (15)	−0.0066 (11)	0.0171 (13)	0.0015 (11)
C17	0.083 (3)	0.0272 (15)	0.0290 (16)	−0.0053 (15)	0.0226 (17)	0.0083 (13)
C18	0.075 (2)	0.0218 (14)	0.0302 (16)	0.0126 (15)	0.0033 (16)	0.0100 (12)
C19	0.0404 (15)	0.0177 (12)	0.0280 (14)	0.0108 (11)	0.0058 (12)	0.0030 (11)
C20	0.0290 (13)	0.0130 (11)	0.0209 (12)	−0.0007 (9)	0.0072 (10)	−0.0001 (9)
C21	0.0231 (14)	0.0267 (15)	0.073 (2)	0.0011 (11)	0.0160 (15)	0.0105 (15)
C22	0.0359 (15)	0.0291 (14)	0.0299 (15)	−0.0080 (12)	0.0041 (12)	0.0064 (12)
C23	0.0366 (16)	0.0152 (13)	0.072 (2)	0.0037 (11)	0.0158 (16)	0.0076 (14)
C24	0.070 (2)	0.042 (2)	0.054 (2)	−0.0220 (18)	−0.0121 (19)	0.0341 (18)
C25	0.079 (3)	0.0366 (18)	0.053 (2)	−0.0262 (18)	0.045 (2)	−0.0056 (16)
C26	0.0362 (14)	0.0185 (12)	0.0326 (15)	0.0019 (10)	0.0189 (12)	0.0101 (11)
C27	0.0395 (16)	0.0289 (14)	0.0458 (18)	−0.0005 (12)	0.0294 (14)	0.0069 (13)
C28	0.0216 (14)	0.0340 (15)	0.061 (2)	0.0054 (11)	0.0183 (14)	−0.0007 (15)
C29	0.0176 (12)	0.0309 (14)	0.0404 (17)	0.0037 (10)	0.0027 (12)	0.0043 (12)
C30	0.0208 (12)	0.0166 (11)	0.0290 (14)	−0.0013 (9)	0.0090 (10)	0.0041 (10)
P1	0.0506 (5)	0.0761 (6)	0.0418 (5)	0.0398 (5)	0.0252 (4)	0.0293 (5)
F1	0.101 (2)	0.136 (2)	0.0600 (15)	0.0695 (18)	0.0506 (15)	0.0277 (16)
F2	0.0968 (18)	0.132 (2)	0.0508 (13)	0.0788 (17)	0.0438 (13)	0.0416 (15)
F3	0.0510 (15)	0.205 (4)	0.076 (2)	0.0419 (19)	0.0156 (14)	0.014 (2)
F4	0.0610 (15)	0.108 (2)	0.0796 (18)	0.0221 (14)	0.0262 (13)	0.0368 (16)
F5	0.134 (2)	0.0842 (18)	0.092 (2)	0.0295 (17)	0.0632 (19)	0.0511 (16)
F6	0.192 (4)	0.111 (2)	0.102 (2)	0.091 (3)	0.067 (2)	0.075 (2)
P2	0.0680 (6)	0.0642 (6)	0.0458 (5)	0.0353 (5)	0.0347 (5)	0.0346 (5)
F11	0.204 (4)	0.137 (3)	0.114 (3)	0.113 (3)	0.071 (3)	0.095 (2)
F12	0.0914 (17)	0.0751 (15)	0.0558 (13)	0.0503 (13)	0.0336 (13)	0.0318 (12)
N1	0.095 (6)	0.114 (6)	0.053 (4)	0.060 (5)	0.044 (4)	0.046 (4)
C31	0.075 (8)	0.086 (6)	0.041 (6)	0.045 (6)	0.037 (5)	0.036 (5)
C32	0.066 (9)	0.117 (9)	0.063 (7)	0.042 (9)	0.035 (8)	0.040 (6)
N1A	0.046 (3)	0.070 (4)	0.050 (4)	0.020 (3)	0.020 (3)	0.031 (3)
C31A	0.051 (4)	0.053 (4)	0.035 (4)	0.012 (3)	0.017 (3)	0.019 (3)
C32A	0.103 (18)	0.103 (9)	0.089 (16)	0.036 (15)	0.077 (16)	0.036 (11)
F7	0.150 (7)	0.212 (10)	0.061 (4)	0.111 (6)	0.079 (5)	0.079 (6)
F8	0.104 (4)	0.122 (7)	0.058 (4)	0.084 (4)	0.056 (4)	0.039 (5)
F9	0.069 (3)	0.107 (4)	0.076 (4)	−0.009 (3)	0.009 (3)	0.022 (3)
F10	0.081 (3)	0.114 (4)	0.122 (5)	−0.002 (3)	0.019 (4)	0.077 (4)
F7A	0.134 (12)	0.122 (11)	0.199 (17)	0.046 (9)	0.120 (13)	0.114 (14)
F8A	0.070 (8)	0.29 (2)	0.22 (2)	0.032 (10)	0.076 (13)	0.17 (2)
F9A	0.211 (19)	0.061 (6)	0.050 (6)	0.051 (8)	0.008 (10)	0.012 (5)
F10A	0.25 (3)	0.133 (17)	0.041 (8)	0.141 (19)	0.021 (12)	0.007 (8)

### Geometric parameters (Å, º)

**Table:**

Hg1—Se3	2.5451 (3)	C12—H12	0.9500
Hg1—Se1	2.5476 (3)	C13—C14	1.404 (5)
Hg1—Se2	2.6254 (3)	C13—H13	0.9500
Hg1—Se2^i^	3.1537 (4)	C14—C15	1.413 (5)
Se1—C10	1.894 (3)	C14—H14	0.9500
Se2—C20	1.895 (3)	C15—H15	0.9500
Se2—Hg1^i^	3.1537 (4)	C16—C17	1.417 (5)
Se3—C30	1.886 (3)	C16—C20	1.427 (4)
Co1—C6	2.018 (3)	C16—H16	0.9500
Co1—C1	2.019 (3)	C17—C18	1.411 (5)
Co1—C7	2.019 (3)	C17—H17	0.9500
Co1—C8	2.024 (3)	C18—C19	1.423 (4)
Co1—C5	2.029 (3)	C18—H18	0.9500
Co1—C2	2.030 (3)	C19—C20	1.426 (4)
Co1—C4	2.030 (3)	C19—H19	0.9500
Co1—C3	2.033 (3)	C21—C25	1.380 (5)
Co1—C9	2.035 (2)	C21—C22	1.390 (4)
Co1—C10	2.056 (2)	C21—H21	0.9500
Co2—C17	2.013 (3)	C22—C23	1.395 (4)
Co2—C18	2.019 (3)	C22—H22	0.9500
Co2—C13	2.023 (3)	C23—C24	1.398 (6)
Co2—C14	2.030 (3)	C23—H23	0.9500
Co2—C12	2.031 (3)	C24—C25	1.405 (6)
Co2—C16	2.034 (3)	C24—H24	0.9500
Co2—C15	2.035 (3)	C25—H25	0.9500
Co2—C11	2.037 (3)	C26—C27	1.418 (4)
Co2—C19	2.041 (3)	C26—C30	1.425 (4)
Co2—C20	2.075 (2)	C26—H26	0.9500
Co3—C25	2.006 (3)	C27—C28	1.411 (5)
Co3—C28	2.008 (3)	C27—H27	0.9500
Co3—C24	2.010 (3)	C28—C29	1.422 (4)
Co3—C27	2.015 (3)	C28—H28	0.9500
Co3—C29	2.021 (3)	C29—C30	1.435 (4)
Co3—C21	2.025 (3)	C29—H29	0.9500
Co3—C23	2.032 (3)	P1—F3	1.559 (3)
Co3—C26	2.039 (2)	P1—F1	1.571 (2)
Co3—C22	2.039 (3)	P1—F4	1.583 (3)
Co3—C30	2.081 (2)	P1—F5	1.584 (3)
C1—C5	1.397 (5)	P1—F2	1.585 (2)
C1—C2	1.408 (5)	P1—F6	1.595 (3)
C1—H1	0.9500	P2—F7A	1.471 (11)
C2—C3	1.399 (5)	P2—F9	1.505 (6)
C2—H2	0.9500	P2—F10A	1.54 (2)
C3—C4	1.423 (5)	P2—F8	1.553 (8)
C3—H3	0.9500	P2—F11	1.566 (3)
C4—C5	1.402 (6)	P2—F7	1.570 (5)
C4—H4	0.9500	P2—F12	1.585 (2)
C5—H5	0.9500	P2—F9A	1.621 (12)
C6—C7	1.418 (4)	P2—F8A	1.642 (14)
C6—C10	1.432 (4)	P2—F10	1.642 (6)
C6—H6	0.9500	N1—C31	1.131 (12)
C7—C8	1.406 (4)	C31—C32	1.427 (16)
C7—H7	0.9500	C32—H32A	0.9800
C8—C9	1.425 (4)	C32—H32B	0.9800
C8—H8	0.9500	C32—H32C	0.9800
C9—C10	1.422 (4)	N1A—C31A	1.126 (9)
C9—H9	0.9500	N1A—N1A^ii^	1.345 (12)
C11—C12	1.400 (5)	C31A—C32A	1.440 (15)
C11—C15	1.413 (5)	C32A—H32D	0.9800
C11—H11	0.9500	C32A—H32E	0.9800
C12—C13	1.407 (5)	C32A—H32F	0.9800
			
Se3—Hg1—Se1	124.083 (9)	Co1—C8—H8	126.4
Se3—Hg1—Se2	116.198 (10)	C10—C9—C8	108.5 (2)
Se1—Hg1—Se2	115.319 (10)	C10—C9—Co1	70.43 (14)
Se3—Hg1—Se2^i^	99.723 (10)	C8—C9—Co1	69.04 (14)
Se1—Hg1—Se2^i^	99.201 (9)	C10—C9—H9	125.8
Se2—Hg1—Se2^i^	91.509 (10)	C8—C9—H9	125.8
C10—Se1—Hg1	104.54 (8)	Co1—C9—H9	126.4
C20—Se2—Hg1	106.04 (7)	C9—C10—C6	106.6 (2)
C20—Se2—Hg1^i^	98.79 (7)	C9—C10—Se1	129.78 (19)
Hg1—Se2—Hg1^i^	88.491 (10)	C6—C10—Se1	123.6 (2)
C30—Se3—Hg1	102.46 (8)	C9—C10—Co1	68.88 (14)
C6—Co1—C1	106.10 (13)	C6—C10—Co1	68.01 (14)
C6—Co1—C7	41.12 (12)	Se1—C10—Co1	126.55 (12)
C1—Co1—C7	113.44 (14)	C12—C11—C15	107.9 (3)
C6—Co1—C8	68.77 (12)	C12—C11—Co2	69.63 (17)
C1—Co1—C8	146.55 (13)	C15—C11—Co2	69.62 (17)
C7—Co1—C8	40.69 (12)	C12—C11—H11	126.0
C6—Co1—C5	113.78 (14)	C15—C11—H11	126.0
C1—Co1—C5	40.39 (14)	Co2—C11—H11	126.3
C7—Co1—C5	145.45 (15)	C11—C12—C13	108.3 (3)
C8—Co1—C5	172.73 (14)	C11—C12—Co2	70.10 (16)
C6—Co1—C2	129.59 (13)	C13—C12—Co2	69.38 (16)
C1—Co1—C2	40.71 (13)	C11—C12—H12	125.8
C7—Co1—C2	107.44 (12)	C13—C12—H12	125.8
C8—Co1—C2	115.98 (12)	Co2—C12—H12	126.3
C5—Co1—C2	68.15 (12)	C14—C13—C12	108.0 (3)
C6—Co1—C4	146.87 (15)	C14—C13—Co2	69.98 (16)
C1—Co1—C4	68.11 (16)	C12—C13—Co2	70.02 (16)
C7—Co1—C4	171.93 (15)	C14—C13—H13	126.0
C8—Co1—C4	134.13 (16)	C12—C13—H13	126.0
C5—Co1—C4	40.42 (16)	Co2—C13—H13	125.6
C2—Co1—C4	68.22 (14)	C13—C14—C15	108.0 (3)
C6—Co1—C3	169.18 (13)	C13—C14—Co2	69.47 (16)
C1—Co1—C3	68.23 (15)	C15—C14—Co2	69.88 (16)
C7—Co1—C3	131.41 (13)	C13—C14—H14	126.0
C8—Co1—C3	110.49 (13)	C15—C14—H14	126.0
C5—Co1—C3	68.37 (14)	Co2—C14—H14	126.2
C2—Co1—C3	40.27 (14)	C14—C15—C11	107.7 (3)
C4—Co1—C3	40.99 (15)	C14—C15—Co2	69.44 (17)
C6—Co1—C9	68.73 (11)	C11—C15—Co2	69.78 (16)
C1—Co1—C9	169.81 (12)	C14—C15—H15	126.1
C7—Co1—C9	68.91 (11)	C11—C15—H15	126.1
C8—Co1—C9	41.10 (10)	Co2—C15—H15	126.2
C5—Co1—C9	132.51 (13)	C17—C16—C20	108.3 (3)
C2—Co1—C9	149.25 (12)	C17—C16—Co2	68.69 (17)
C4—Co1—C9	111.05 (14)	C20—C16—Co2	71.22 (14)
C3—Co1—C9	118.31 (13)	C17—C16—H16	125.9
C6—Co1—C10	41.14 (10)	C20—C16—H16	125.9
C1—Co1—C10	129.79 (12)	Co2—C16—H16	125.8
C7—Co1—C10	69.29 (10)	C18—C17—C16	108.2 (3)
C8—Co1—C10	68.99 (10)	C18—C17—Co2	69.77 (18)
C5—Co1—C10	108.10 (11)	C16—C17—Co2	70.33 (16)
C2—Co1—C10	168.83 (12)	C18—C17—H17	125.9
C4—Co1—C10	116.29 (12)	C16—C17—H17	125.9
C3—Co1—C10	149.47 (13)	Co2—C17—H17	125.6
C9—Co1—C10	40.70 (10)	C17—C18—C19	108.2 (3)
C17—Co2—C18	40.97 (15)	C17—C18—Co2	69.26 (17)
C17—Co2—C13	104.66 (13)	C19—C18—Co2	70.27 (16)
C18—Co2—C13	118.79 (13)	C17—C18—H18	125.9
C17—Co2—C14	122.20 (14)	C19—C18—H18	125.9
C18—Co2—C14	106.12 (13)	Co2—C18—H18	126.1
C13—Co2—C14	40.55 (13)	C18—C19—C20	108.0 (3)
C17—Co2—C12	119.40 (14)	C18—C19—Co2	68.68 (16)
C18—Co2—C12	154.38 (14)	C20—C19—Co2	71.02 (14)
C13—Co2—C12	40.60 (13)	C18—C19—H19	126.0
C14—Co2—C12	68.12 (12)	C20—C19—H19	126.0
C17—Co2—C16	40.98 (13)	Co2—C19—H19	125.9
C18—Co2—C16	68.82 (14)	C19—C20—C16	107.3 (2)
C13—Co2—C16	122.91 (12)	C19—C20—Se2	128.4 (2)
C14—Co2—C16	159.32 (13)	C16—C20—Se2	124.3 (2)
C12—Co2—C16	107.28 (12)	C19—C20—Co2	68.43 (14)
C17—Co2—C15	160.25 (15)	C16—C20—Co2	68.17 (14)
C18—Co2—C15	124.82 (15)	Se2—C20—Co2	128.02 (12)
C13—Co2—C15	68.31 (13)	C25—C21—C22	108.4 (3)
C14—Co2—C15	40.68 (14)	C25—C21—Co3	69.25 (19)
C12—Co2—C15	68.04 (12)	C22—C21—Co3	70.55 (16)
C16—Co2—C15	158.24 (14)	C25—C21—H21	125.8
C17—Co2—C11	155.77 (15)	C22—C21—H21	125.8
C18—Co2—C11	162.92 (15)	Co3—C21—H21	126.0
C13—Co2—C11	68.18 (13)	C21—C22—C23	108.1 (3)
C14—Co2—C11	68.27 (13)	C21—C22—Co3	69.44 (17)
C12—Co2—C11	40.27 (13)	C23—C22—Co3	69.68 (16)
C16—Co2—C11	122.15 (13)	C21—C22—H22	125.9
C15—Co2—C11	40.60 (13)	C23—C22—H22	125.9
C17—Co2—C19	69.01 (13)	Co3—C22—H22	126.5
C18—Co2—C19	41.04 (12)	C22—C23—C24	107.8 (3)
C13—Co2—C19	155.45 (12)	C22—C23—Co3	70.25 (16)
C14—Co2—C19	121.50 (12)	C24—C23—Co3	68.93 (18)
C12—Co2—C19	163.16 (12)	C22—C23—H23	126.1
C16—Co2—C19	68.67 (12)	C24—C23—H23	126.1
C15—Co2—C19	109.28 (12)	Co3—C23—H23	126.3
C11—Co2—C19	126.94 (12)	C23—C24—C25	107.6 (3)
C17—Co2—C20	68.61 (11)	C23—C24—Co3	70.62 (18)
C18—Co2—C20	68.51 (11)	C25—C24—Co3	69.38 (18)
C13—Co2—C20	161.03 (12)	C23—C24—H24	126.2
C14—Co2—C20	158.00 (12)	C25—C24—H24	126.2
C12—Co2—C20	125.96 (11)	Co3—C24—H24	125.4
C16—Co2—C20	40.62 (10)	C21—C25—C24	108.1 (3)
C15—Co2—C20	123.73 (12)	C21—C25—Co3	70.72 (18)
C11—Co2—C20	110.28 (11)	C24—C25—Co3	69.7 (2)
C19—Co2—C20	40.55 (10)	C21—C25—H25	125.9
C25—Co3—C28	151.76 (18)	C24—C25—H25	125.9
C25—Co3—C24	40.96 (17)	Co3—C25—H25	125.3
C28—Co3—C24	117.02 (17)	C27—C26—C30	108.8 (3)
C25—Co3—C27	166.20 (17)	C27—C26—Co3	68.63 (15)
C28—Co3—C27	41.05 (14)	C30—C26—Co3	71.34 (14)
C24—Co3—C27	151.55 (17)	C27—C26—H26	125.6
C25—Co3—C29	117.88 (15)	C30—C26—H26	125.6
C28—Co3—C29	41.32 (13)	Co3—C26—H26	126.0
C24—Co3—C29	106.39 (14)	C28—C27—C26	108.1 (3)
C27—Co3—C29	69.23 (13)	C28—C27—Co3	69.21 (17)
C25—Co3—C21	40.03 (16)	C26—C27—Co3	70.43 (15)
C28—Co3—C21	165.87 (15)	C28—C27—H27	125.9
C24—Co3—C21	67.94 (15)	C26—C27—H27	125.9
C27—Co3—C21	128.78 (15)	Co3—C27—H27	126.0
C29—Co3—C21	152.40 (13)	C27—C28—C29	108.1 (3)
C25—Co3—C23	68.09 (14)	C27—C28—Co3	69.74 (16)
C28—Co3—C23	106.95 (12)	C29—C28—Co3	69.80 (16)
C24—Co3—C23	40.45 (16)	C27—C28—H28	126.0
C27—Co3—C23	118.42 (13)	C29—C28—H28	126.0
C29—Co3—C23	126.63 (12)	Co3—C28—H28	126.1
C21—Co3—C23	67.55 (12)	C28—C29—C30	108.4 (3)
C25—Co3—C26	128.18 (14)	C28—C29—Co3	68.88 (16)
C28—Co3—C26	68.92 (12)	C30—C29—Co3	71.79 (14)
C24—Co3—C26	165.64 (16)	C28—C29—H29	125.8
C27—Co3—C26	40.94 (11)	C30—C29—H29	125.8
C29—Co3—C26	68.77 (12)	Co3—C29—H29	125.1
C21—Co3—C26	109.67 (12)	C26—C30—C29	106.6 (2)
C23—Co3—C26	153.11 (14)	C26—C30—Se3	130.28 (19)
C25—Co3—C22	67.48 (14)	C29—C30—Se3	123.1 (2)
C28—Co3—C22	127.60 (13)	C26—C30—Co3	68.20 (14)
C24—Co3—C22	67.71 (13)	C29—C30—Co3	67.29 (14)
C27—Co3—C22	108.91 (13)	Se3—C30—Co3	129.52 (12)
C29—Co3—C22	164.94 (12)	F3—P1—F1	90.82 (16)
C21—Co3—C22	40.01 (13)	F3—P1—F4	177.97 (19)
C23—Co3—C22	40.07 (13)	F1—P1—F4	90.61 (16)
C26—Co3—C22	120.28 (12)	F3—P1—F5	94.2 (2)
C25—Co3—C30	108.22 (12)	F1—P1—F5	90.20 (16)
C28—Co3—C30	68.96 (11)	F4—P1—F5	87.26 (16)
C24—Co3—C30	127.29 (14)	F3—P1—F2	88.73 (16)
C27—Co3—C30	68.69 (11)	F1—P1—F2	179.55 (15)
C29—Co3—C30	40.91 (10)	F4—P1—F2	89.84 (15)
C21—Co3—C30	119.73 (11)	F5—P1—F2	89.87 (15)
C23—Co3—C30	164.93 (13)	F3—P1—F6	88.3 (2)
C26—Co3—C30	40.46 (10)	F1—P1—F6	90.27 (17)
C22—Co3—C30	153.56 (11)	F4—P1—F6	90.23 (19)
C5—C1—C2	108.3 (3)	F5—P1—F6	177.5 (2)
C5—C1—Co1	70.19 (18)	F2—P1—F6	89.67 (16)
C2—C1—Co1	70.06 (16)	F7A—P2—F10A	97.8 (13)
C5—C1—H1	125.9	F9—P2—F8	92.2 (5)
C2—C1—H1	125.9	F7A—P2—F11	77.8 (6)
Co1—C1—H1	125.5	F9—P2—F11	87.0 (3)
C3—C2—C1	108.2 (3)	F10A—P2—F11	84.2 (10)
C3—C2—Co1	70.01 (17)	F8—P2—F11	91.9 (4)
C1—C2—Co1	69.24 (16)	F9—P2—F7	92.0 (4)
C3—C2—H2	125.9	F8—P2—F7	175.3 (5)
C1—C2—H2	125.9	F11—P2—F7	90.6 (3)
Co1—C2—H2	126.4	F7A—P2—F12	99.1 (6)
C2—C3—C4	107.6 (3)	F9—P2—F12	96.2 (3)
C2—C3—Co1	69.72 (17)	F10A—P2—F12	95.3 (10)
C4—C3—Co1	69.39 (18)	F8—P2—F12	88.6 (4)
C2—C3—H3	126.2	F11—P2—F12	176.7 (2)
C4—C3—H3	126.2	F7—P2—F12	88.7 (3)
Co1—C3—H3	126.3	F7A—P2—F9A	92.5 (9)
C5—C4—C3	107.8 (3)	F10A—P2—F9A	169.3 (13)
C5—C4—Co1	69.73 (19)	F11—P2—F9A	95.3 (5)
C3—C4—Co1	69.62 (18)	F12—P2—F9A	85.9 (5)
C5—C4—H4	126.1	F7A—P2—F8A	175.2 (11)
C3—C4—H4	126.1	F10A—P2—F8A	86.6 (13)
Co1—C4—H4	126.1	F11—P2—F8A	100.8 (7)
C1—C5—C4	108.2 (3)	F12—P2—F8A	82.4 (7)
C1—C5—Co1	69.43 (17)	F9A—P2—F8A	83.0 (10)
C4—C5—Co1	69.85 (18)	F9—P2—F10	177.7 (3)
C1—C5—H5	125.9	F8—P2—F10	87.0 (5)
C4—C5—H5	125.9	F11—P2—F10	95.2 (3)
Co1—C5—H5	126.4	F7—P2—F10	88.7 (4)
C7—C6—C10	108.8 (2)	F12—P2—F10	81.5 (2)
C7—C6—Co1	69.48 (16)	N1—C31—C32	176.8 (19)
C10—C6—Co1	70.84 (14)	C31—C32—H32A	109.5
C7—C6—H6	125.6	C31—C32—H32B	109.5
C10—C6—H6	125.6	H32A—C32—H32B	109.5
Co1—C6—H6	125.6	C31—C32—H32C	109.5
C8—C7—C6	107.9 (2)	H32A—C32—H32C	109.5
C8—C7—Co1	69.85 (15)	H32B—C32—H32C	109.5
C6—C7—Co1	69.40 (15)	C31A—N1A—N1A^ii^	163.0 (9)
C8—C7—H7	126.0	N1A—C31A—C32A	179.9 (11)
C6—C7—H7	126.0	C31A—C32A—H32D	109.5
Co1—C7—H7	126.3	C31A—C32A—H32E	109.5
C7—C8—C9	108.3 (2)	H32D—C32A—H32E	109.5
C7—C8—Co1	69.46 (15)	C31A—C32A—H32F	109.5
C9—C8—Co1	69.87 (14)	H32D—C32A—H32F	109.5
C7—C8—H8	125.9	H32E—C32A—H32F	109.5
C9—C8—H8	125.9		
			
C5—C1—C2—C3	0.6 (3)	C16—C17—C18—Co2	−60.1 (2)
Co1—C1—C2—C3	−59.4 (2)	C17—C18—C19—C20	1.4 (3)
C5—C1—C2—Co1	60.0 (2)	Co2—C18—C19—C20	60.42 (18)
C1—C2—C3—C4	−0.4 (3)	C17—C18—C19—Co2	−59.1 (2)
Co1—C2—C3—C4	−59.3 (2)	C18—C19—C20—C16	−1.8 (3)
C1—C2—C3—Co1	58.9 (2)	Co2—C19—C20—C16	57.16 (17)
C2—C3—C4—C5	0.0 (3)	C18—C19—C20—Se2	178.91 (19)
Co1—C3—C4—C5	−59.5 (2)	Co2—C19—C20—Se2	−122.13 (19)
C2—C3—C4—Co1	59.5 (2)	C18—C19—C20—Co2	−58.96 (18)
C2—C1—C5—C4	−0.6 (3)	C17—C16—C20—C19	1.6 (3)
Co1—C1—C5—C4	59.3 (2)	Co2—C16—C20—C19	−57.32 (17)
C2—C1—C5—Co1	−59.9 (2)	C17—C16—C20—Se2	−179.09 (18)
C3—C4—C5—C1	0.4 (3)	Co2—C16—C20—Se2	122.00 (17)
Co1—C4—C5—C1	−59.0 (2)	C17—C16—C20—Co2	58.90 (18)
C3—C4—C5—Co1	59.5 (2)	Hg1—Se2—C20—C19	−33.8 (2)
C10—C6—C7—C8	−0.7 (3)	Hg1^i^—Se2—C20—C19	−124.7 (2)
Co1—C6—C7—C8	59.45 (19)	Hg1—Se2—C20—C16	147.06 (19)
C10—C6—C7—Co1	−60.18 (18)	Hg1^i^—Se2—C20—C16	56.1 (2)
C6—C7—C8—C9	0.1 (3)	Hg1—Se2—C20—Co2	−125.16 (14)
Co1—C7—C8—C9	59.30 (18)	Hg1^i^—Se2—C20—Co2	143.88 (14)
C6—C7—C8—Co1	−59.17 (18)	C25—C21—C22—C23	0.0 (3)
C7—C8—C9—C10	0.5 (3)	Co3—C21—C22—C23	−59.1 (2)
Co1—C8—C9—C10	59.57 (17)	C25—C21—C22—Co3	59.1 (2)
C7—C8—C9—Co1	−59.04 (18)	C21—C22—C23—C24	0.0 (3)
C8—C9—C10—C6	−1.0 (3)	Co3—C22—C23—C24	−58.9 (2)
Co1—C9—C10—C6	57.75 (17)	C21—C22—C23—Co3	59.0 (2)
C8—C9—C10—Se1	−179.32 (18)	C22—C23—C24—C25	−0.1 (3)
Co1—C9—C10—Se1	−120.6 (2)	Co3—C23—C24—C25	−59.8 (2)
C8—C9—C10—Co1	−58.71 (17)	C22—C23—C24—Co3	59.8 (2)
C7—C6—C10—C9	1.0 (3)	C22—C21—C25—C24	0.0 (3)
Co1—C6—C10—C9	−58.30 (17)	Co3—C21—C25—C24	59.9 (2)
C7—C6—C10—Se1	179.53 (17)	C22—C21—C25—Co3	−59.9 (2)
Co1—C6—C10—Se1	120.19 (17)	C23—C24—C25—C21	0.1 (4)
C7—C6—C10—Co1	59.34 (18)	Co3—C24—C25—C21	−60.5 (2)
Hg1—Se1—C10—C9	−8.8 (2)	C23—C24—C25—Co3	60.6 (2)
Hg1—Se1—C10—C6	173.14 (19)	C30—C26—C27—C28	−1.1 (3)
Hg1—Se1—C10—Co1	−100.68 (14)	Co3—C26—C27—C28	59.16 (19)
C15—C11—C12—C13	0.3 (3)	C30—C26—C27—Co3	−60.26 (17)
Co2—C11—C12—C13	−59.0 (2)	C26—C27—C28—C29	−0.4 (3)
C15—C11—C12—Co2	59.29 (19)	Co3—C27—C28—C29	59.5 (2)
C11—C12—C13—C14	−0.4 (3)	C26—C27—C28—Co3	−59.92 (18)
Co2—C12—C13—C14	−59.9 (2)	C27—C28—C29—C30	1.8 (3)
C11—C12—C13—Co2	59.48 (19)	Co3—C28—C29—C30	61.25 (18)
C12—C13—C14—C15	0.4 (3)	C27—C28—C29—Co3	−59.45 (19)
Co2—C13—C14—C15	−59.5 (2)	C27—C26—C30—C29	2.2 (3)
C12—C13—C14—Co2	59.93 (19)	Co3—C26—C30—C29	−56.42 (17)
C13—C14—C15—C11	−0.3 (3)	C27—C26—C30—Se3	−177.39 (18)
Co2—C14—C15—C11	−59.51 (19)	Co3—C26—C30—Se3	124.0 (2)
C13—C14—C15—Co2	59.3 (2)	C27—C26—C30—Co3	58.60 (17)
C12—C11—C15—C14	0.0 (3)	C28—C29—C30—C26	−2.4 (3)
Co2—C11—C15—C14	59.3 (2)	Co3—C29—C30—C26	56.98 (17)
C12—C11—C15—Co2	−59.30 (19)	C28—C29—C30—Se3	177.18 (18)
C20—C16—C17—C18	−0.8 (3)	Co3—C29—C30—Se3	−123.41 (17)
Co2—C16—C17—C18	59.7 (2)	C28—C29—C30—Co3	−59.42 (19)
C20—C16—C17—Co2	−60.48 (18)	Hg1—Se3—C30—C26	−2.6 (2)
C16—C17—C18—C19	−0.4 (3)	Hg1—Se3—C30—C29	177.93 (19)
Co2—C17—C18—C19	59.7 (2)	Hg1—Se3—C30—Co3	91.34 (16)

Symmetry codes: (i) −*x*+1, −*y*+1, −*z*+1; (ii) −*x*+2, −*y*+1, −*z*+2.
